# Supplementary material for: Extraction Optimization and Structural Characteristics of Chitosan from Cuttlefish (S. pharaonis sp.) Bone
Source: Materials (Basel). 2022 Nov 11;15(22):7969. doi: 10.3390/ma15227969 (PMC9698347; doi:10.3390/ma15227969)
Supplement: Supplementary file 1 [file materials-15-07969-s001.zip › materials-2000674-supplementary.pdf]

Supplementary

Table S1. Variation of the response surface method, ordinal sum of squares and regression analysis of the three-level factor on the chitosan yield from cuttlefish bones.

**Source**	**Sum of Squares**	**df**	**Mean Square**	**F-value**	**p-value**	
**Model**	555.21	9	61.69	4.79	0.0496	Significant
A-NaOH	18.72	1	18.72	1.45	0.2818	
B-Time	23.26	1	23.26	1.81	0.2366	
C-Temp	28.13	1	28.13	2.19	0.1994	
AB	114.49	1	114.49	8.89	0.0307	Significant
AC	58.34	1	58.34	4.53	0.0865	
BC	107.30	1	107.30	8.34	0.0343	Significant
A^2	118.00	1	118.00	9.17	0.0292	Significant
B^2	99.67	1	99.67	7.74	0.0388	Significant
C^2	45.64	1	45.64	3.55	0.1184	
**Residual**	64.36	5	12.87			
Lack of Fit	61.04	3	20.35	12.24	0.0765	Not significant
Pure Error	3.33	2	1.66			
**Cor Total**	619.57	14				

Std Dev.	Mean	CV%	R^2	Adjusted R^2	Predicted R^2	Adeq Precision
3.59	48.52	7.39	0.8961	0.7091	-0.7002	6.0993
